# Irish Bulls

**Published:** 1880-07

**Authors:** 


### IRISH BULLS.

The *Maryland Medical Journal* for April contains a very interesting article from the pen of Dr. John V. Keating, of Philadelphia, entitled "A pin extracted from the throat of a child three years old," but after reading it we are surprised to find it was "extracted" from the anus—only a change of base.

The report of an Irish Benevolent Society says: "Notwithstanding the large amount paid for medicine and medical attendance, very few deaths occurred during the year."

